# Global solar wind variations over the last four centuries

**DOI:** 10.1038/srep41548

**Published:** 2017-01-31

**Authors:** M. J. Owens, M. Lockwood, P. Riley

**Affiliations:** 1 Space and Atmospheric Electricity Group, Department of Meteorology, University of Reading, Earley Gate, PO Box 243, Reading RG6 6BB, UK; 2 Predictive Science Inc., 9990 Mesa Rim Rd, Suite 170, San Diego, CA 92121, USA

## Abstract

The most recent “grand minimum” of solar activity, the Maunder minimum (MM, 1650–1710), is of great interest both for understanding the solar dynamo and providing insight into possible future heliospheric conditions. Here, we use nearly 30 years of output from a data-constrained magnetohydrodynamic model of the solar corona to calibrate heliospheric reconstructions based solely on sunspot observations. Using these empirical relations, we produce the first quantitative estimate of global solar wind variations over the last 400 years. Relative to the modern era, the MM shows a factor 2 reduction in near-Earth heliospheric magnetic field strength and solar wind speed, and up to a factor 4 increase in solar wind Mach number. Thus solar wind energy input into the Earth’s magnetosphere was reduced, resulting in a more Jupiter-like system, in agreement with the dearth of auroral reports from the time. The global heliosphere was both smaller and more symmetric under MM conditions, which has implications for the interpretation of cosmogenic radionuclide data and resulting total solar irradiance estimates during grand minima.

The structure of the solar wind, and of the magnetic field it drags from the Sun to form the heliosphere, varies in a fundamental way with the phase of the sunspot cycle. At sunspot minimum, the solar wind is highly structured by solar latitude. Fast, tenuous solar wind originates from polar coronal holes associated with “open” magnetic flux^1^ and slower, denser solar wind arises from equatorial streamer belts associated with closed magnetic loops^2^. At sunspot maximum regions of fast and slow wind are found at all latitudes^3^,^4^ and the corona is much more dynamic as a result of increased coronal mass ejection activity^5^.

Direct observations of the solar wind and heliospheric magnetic field (HMF) outside the ecliptic plane are primarily limited to single-point *in*-*situ* measurements taken by the solar polar-orbiting Ulysses mission^3^, 1991–2008. Interplanetary scintillation (routinely performed since circa 1989)^6^ can infer the solar wind density and speed integrated along the line-of-sight to suitable astrophysical radio sources and, when combined with tomographic techniques, can provide greater spatial sampling than *in*-*situ* observations, though with greater uncertainty. Observationally constrained coronal modelling, particularly photospheric magnetogram extrapolation (routinely possible since 1975)^7^,^8^,^9^, provides global estimates of HMF and, indirectly, solar wind structure. The accuracy of such estimates depends on both the underlying observations and the model assumptions and at present they are more reliable at solar minimum than at solar maximum^10^. Finally, coronagraph and eclipse observations reveal coronal density structures and hence can give indirect insight into the solar wind structure.

The local heliosphere in near-Earth space has been directly sampled for the last 60 years^11^, while geomagnetic proxies can be used to reliably infer annual means of HMF and solar wind speed back to 1845^12^,^13^. Unfortunately, even this extended interval does not contain a “grand solar minimum” of activity, the most recent of which was the Maunder minimum (MM), circa 1650–1710, when there were a dearth of sunspots and a greatly reduced occurrence of reported aurora^14^,^15^,^16^. The MM is of great interest to solar dynamo theory and modelling^17^, as well as for constraining future heliospheric variations, particularly the space-weather implications of a possible long-term decline of solar activity^18^. Quantitative knowledge of the MM is limited to sunspot records (which extend back to 1610)^19^ and indirect estimates of the HMF from cosmogenic radionuclides^20^, such as ^10^Be in ice cores or ^14^C in tree trunks^21^,^22^,^23^.

Sunspot records can be used to estimate the total magnetic flux that leaves the solar corona and enters the heliosphere, referred to as the open solar flux (OSF), using a continuity equation^24^. New OSF is generated by coronal loops dragged out into the heliosphere by the solar wind, expected to vary non-linearly with sunspot number and hence with both the phase and amplitude of the solar cycle^12^. OSF is lost by near-Sun reconnection, controlled by the local inclination of the heliospheric current sheet (HCS) to the solar rotation direction^25^. As such, OSF loss is found to be a function of only solar cycle phase. Thus OSF can be reconstructed by sunspot number alone. Such estimates of OSF have been well validated against geomagnetic reconstructions back to 1845^12^ and against ^10^Be, ^14^C and ^44^Ti in meteoritic material back to 1610^15^,^26^.

New OSF, in the form of closed loops, will enter the streamer belt. As these loops propagate further into the heliosphere, they will ultimately add to the open corona-hole flux. By optimising the time constant for conversion between streamer belt and coronal hole flux, Lockwood and Owens^27^ reconstructed the fraction of OSF contained within the streamer belt. From this, they estimated the latitudinal streamer belt width (SBW) as SBW = sin^−1^ (F_SB_/OSF), where F_SB_ is the magnetic flux contained in the streamer belt, back to 1617. The observed correspondence between slow wind and the streamer belt means SBW is expected to be closely related to the latitudinal extent of slow solar wind. Figure 1 summarises the pertinent results of Lockwood and Owens: panel a shows the two sunspot data composites considered; R_G_, the group sunspot number record^19^, and R_C_, a composite of group and international records with various calibrations/corrections applied^28^. Panel b shows the estimate of SBW from the sunspot records and their agreement with SBW estimates manually scaled from available eclipse images (detailed in the Figure caption and Appendix A). It can be seen while the two sunspot records differ considerably, the resulting SBW is not sensitive to the choice of sunspot record. In particular, the change in SBW between the modern era (e.g., 1960-present) and the MM is very similar for the two sunspot number records. Thus while there is currently much on-going work to recalibrate and correct the sunspot records^29^,^30^,^31^, the outcomes are not expected to greatly influence the results presented here.

## Methods

SBW is now calibrated in terms of solar wind speed, V_SW_, using a combination of *in situ* spacecraft observations and the “Magnetohydrodynamics Around a Sphere” (MAS^32^) global coronal model constrained by photospheric magnetic field observations. For a given Carrington rotation (CR), the MAS model extrapolates the photospheric field distribution outward to 30 solar radii (R_S_), while self-consistently solving the plasma parameters on a non-uniform grid in polar coordinates, using the MHD equations and the vector potential **A** (where the magnetic field, **B**, is given by ∇ × **A**), such that ∇.∇× **A** = 0 (which ensures current continuity, ∇. **J** = 0, is conserved to within the model’s numerical accuracy). All model data can be downloaded from http://www.predsci.com/mhdweb/home.php. The calibration period covers 1975–2013, the timespan for which both photospheric magnetograms (and hence MAS estimates) and SBW estimates are available. A combination of Kitt Peak, Wilcox Solar Observatory, Mount Wilson Solar Observatory, SOLIS and GONG data are used to minimse data gaps and provide the longest possible time sequence. V_SW_ is extracted at 30 R_S_, the interface of the coronal and solar wind models.

Comparison of MAS with spacecraft data reveals MAS reproduces the observed solar wind structure, but generally underestimates the fast wind speed. A linear scaling of 1.04 and an offset of 78.5 km/s is determined to provide the best fit of MAS 27-day means (discussed below) to spacecraft V_SW_ observations. This corrected MAS solar wind speed is referred to as V_MAS_ and shown in Fig. 2. To later enable comparison with the annual sunspot-based SBW estimates, which provide information about the latitudinal solar wind structure, but no longitudinal information, “zonal means” (i.e., longitudinal or 27-day averages) are used. Data are further smoothed using a 1-year rolling (boxcar) means, to mimic the annual SBW estimates while retaining rapid latitudinal variations of the observing spacecraft.

Figure 2a shows sunspot number and the latitude of the observing spacecraft, with the near-Earth OMNI data^11^ in white and Ulysses^33^ in red. Figure 2b shows in-ecliptic V_SW_ observations from OMNI (black) and V_MAS_ (blue) at Earth’s heliographic latitude. There is only a very weak solar-cycle variation annual V_SW_. Indeed, to first order, the OMNI V_SW_ can be approximated as 430 km/s with ~50 km/s variability. In that respect, V_MAS_ is in good agreement. Observations made by the Ulysses spacecraft, shown in Fig. 2c, provide a better picture of global solar wind structure. Ulysses performed 3 “fast-latitude scans” of the Sun, sampling all solar latitude within approximately 1 year. For the two solar minimum fast-latitude scans, in approximately 1994–1996 and 2006–2008, the global solar wind structure is fast wind from the poles and a narrow slow wind band at the equator. This is well reproduced by MAS. The broader slow wind band during the second solar minimum pass (2006–2008), partly the result of increased pseudostreamer occurrence^2^, is also captured by MAS. During the solar maximum fast-latitude scan, 2000–2002, the agreement is good in the northern hemisphere, but V_MAS_ is higher than observed in the southern hemisphere^34^. In general, however, there is sufficient agreement between V_MAS_ and observed V_SW_ that we proceed in using V_MAS_ to calibrate the sunspot-based reconstructions.

An example of V_MAS_ for a single Carrington rotation (CR1756, which approximately spans December 1984, the late declining phase of solar cycle 21), is shown in Fig. 3. Panel a shows V_MAS_ at 30 R_S_ in heliographic coordinates. The data have been reduced in resolution to 90 and 45 equally-spaced grid points in longitude and sine latitude, respectively, to enable efficient manipulation of the data. The heliospheric current sheet (HCS) is shown as the thin white line. The dashed black line in panel b shows the zonal mean V_MAS_ for CR1756. As is typical of non-solar maximum periods, the solar wind structure can be approximated as fast wind from the poles, with a roughly equatorial “belt” of slow wind, primarily centred on the HCS. Thus the zonal mean V_SW_ in heliographic coordinates is a product of a number of factors:

1. The inclination of the slow wind belt to the solar rotation direction. Given the association of slow wind with the HCS, this is to first order equal to the magnetic dipole tilt.
2. The angular width of the slow solar wind belt about the HCS location.
3. The higher order “waviness” of the belt. The waviness of the HCS results from quadrapolar (and higher) order components of the magnetic field.
4. Sources of slow wind not directly associated with the HCS (e.g. pseudostreamers or “S-web”^35^).

As is shown below, factor 1, the large-scale inclination of the solar wind speed structure to the solar equator, is a strong function of solar cycle phase and thus does not need to be calibrated in terms of SBW. Removal of inclination from V_MAS_ estimates allows the true slow wind band width to be more readily estimated from zonal means. To this end, the V_MAS_ solution at 30 R_S_ is put through a coordinate transform to remove the large-scale inclination of the slow wind band. As will be shown, this is approximately equivalent to transforming to heliomagnetic coordinates. Specifically, we determine the coordinate system which maximises the difference in zonal-mean V_MAS_ between the equator and poles. For CR1756, this requires inclining the North rotation pole up by 32 degrees (through a longitude of −74 degrees). V_MAS_ in this inclined coordinate system is shown in panel c. While the coordinate transform is determined purely from V_MAS_ data, it can clearly be seen that it also reduces the overall magnetic dipole tilt, producing a HCS much closer to the equator (though shorter-scale corrugation is still present). Zonal mean V_MAS_ in the inclined coordinate system (panel d) exhibits a narrower and deeper slow wind belt than in heliographic coordinates. Note that angular width of the zonal mean slow wind band in inclined coordinates still includes the waviness of the slow wind belt (factor 3) and the non-HCS sources (factor 4), which we assume can be calibrated in terms of SBW.

Figure 4 shows zonal mean V_MAS_ for all Carrington rotations in the period 1975–2013. Panel a shows data in heliographic coordinates, while panel c shows the inclined coordinate system, as described above. The angle required for the axis inclination is shown in panel b. As expected, the time variation of the inclination angle approximately follows the HCS tilt (e.g., Fig. 4 of Owens and Lockwood^36^) with small values at solar minimum, when the rotation and magnetic axes are aligned, and a saw-tooth increase peaking at solar maximum. As can be seen from panel c, inclined coordinates do generally result in a narrower slow wind band than heliographic coordinates. In particular, slow solar wind at mid-latitudes in heliographic coordinates during the declining phase of the solar cycle (e.g., 2002–2004) is largely absent in inclined coordinates, indicating that the broadened zonal-mean slow wind band is the result of increased tilt of the slow wind band to the solar rotation direction, not a broadening of the slow wind band itself. On the other hand, the increased latitudinal extent of the slow wind during the 2007–2009 solar minimum over with the previous 2 minima appears to be the result of both increased tilt and broadening of the band itself.

The next step is to characterise zonal mean V_MAS_ structure for each CR by a reduced number of parameters. We use a simple functional form for the zonal mean V_MAS_, describing it by a maximum solar wind speed (*V*_*0*_) with a sinusoidal dip, centred on the equator, of depth *dV* and angular width *θ*_*V*_^37^. As SBW contains no hemispheric information, hemispheric averages of zonal V_MAS_ are used, shown as solid black lines in Fig. 3b and d. *V*_*0*_ is taken to be the maximum value of the hemispheric-averaged zonal mean V_MAS_. *dV* is the difference between *V*_*0*_ and the minimum value of the hemispheric-averaged zonal mean V_MAS_. *θ*_*V*_ is fit as a free parameter. Examples of best fits are shown as the red lines in Fig. 3b and d. In heliographic coordinates, CR1756 yields *V*_*0*_ = 757 km/s, *θ*_*V*_ = 61.1 degrees and *dV* = 318 km/s. In inclined coordinates it yields *V*_*0*_ = 757 km/s, *θ*_*V*_ = 34.0 degrees and *dV* = 373 km/s.

The black lines in panels a to c of Fig. 5 show time series of annual means of *V*_*0*_, *θ*_*V*_ and *dV* (from data in inclined coordinates) over the period 1975–2013. In panel a, *θ*_*V*_ exhibits a strong, saw-tooth-like, solar cycle variation, with an increase in *θ*_*V*_ over the last three minima. The 1980 and 2000 maxima show a slower decline in *θ*_*V*_ than the 1990 maximum. *V*_*0*_ is constant at 757 km/s for the bulk of the interval, with short-duration (1–2 years) drops at solar maximum (panel b). This is a result of the prevalence of slow solar wind at all latitudes at solar maximum, both in heliographic and inclined coordinates. *dV* is only weakly ordered by the solar cycle (panel c).

Panels d to f of Fig. 5 show scatter plots of *V*_*0*_, *θ*_*V*_ and *dV*, respectively, with the SBW estimates from the sunspot-based OSF model. Panel d shows that SBW is strongly ordered with *θ*_*V*_, as expected. The red line shows the 2^nd^ order polynomial fit, which is used to produce the *θ*_*V*_ estimate from SBW (shown as the red line in panel a). The agreement is very good (linear correlation coefficient, r = 0.86 for N = 36), with both the solar cycle and cycle-to-cycle variations well captured. Panel e shows a more complex relation between *V*_*0*_ and SBW. For low values of SBW, *V*_*0*_ shows a constant value of 757 km/s. For high values of SBW, however, there is an approximately linear fall off in *V*_*0*_. Fitting these two regimes separately yields the *V*_*0*_ reconstruction shown as a red line in panel (b). Both the solar cycle and cycle-to-cycle trends in *V*_*0*_ are well captured and the correlation is strong (r = 0.71, N = 36). Finally, panel f shows *dV* and SBW. Correlation is weak (r = 0.32, N = 36), though a statistically significant downward trend is present, shown by the red line. The associated *dV* reconstruction is shown in panel c. Agreement is relatively poor, though as will be shown in the next section, this does not greatly affect the reconstruction of zonal mean V_SW_.

## Results

The three relations shown in Fig. 5 are used to reconstruct annual zonal mean V_SW_ in inclined coordinates on the basis of sunspot-based SBW estimates. The inclination angle is assumed to be a function only of solar-cycle phase, as shown by the red line in Fig. 4b. The resulting V_SW_ reconstruction in heliographic coordinates is referred to as V_RECON_. Figure 2 shows a comparison of V_RECON_ at Earth’s heliographic latitude with both *in situ* V_SW_ observations and V_MAS_. As expected, the exact details of in-ecliptic solar wind speed (panel b) are not captured, but V_RECON_ produces the approximately the same mean and range of variability as observed. The global solar wind structure revealed by Ulysses (panel c) is well matched, with a notable exception: As with V_MAS_, faster wind is present over the south pole around 2000 than was observed.

In order to investigate the global structure of V_RECON_ further, it is compared directly with V_MAS_ in Fig. 6. Panel a shows annual means of V_RECON_ (top) and V_MAS_ (bottom) as a function of heliographic latitude and time. In general, the agreement is very good, both in the solar cycle variation and the cycle-to-cycle variations, particularly the broader slow solar wind band in the 2007–2010 minimum compared with the previous minima. There are, however, a number of differences. Firstly, the slow wind band broadening at solar maximum (approximately 1980, 1990 and 2000) occurs around 0.5 to 1 year earlier in V_RECON_ than V_MAS_. Secondly, during the declining phase of the solar cycle, particularly 1992–1994, the slow wind band narrows more rapidly in V_RECON_ than in V_MAS_. Figure 6b shows the global mean solar wind speed from a latitudinal integration of the data presented in Fig. 6a (allowing for the reduced element area towards the poles). Again, the general agreement is very good (r = 0.87, N = 39). While the approximately 0.5- to 1-year time lag is present for much of 1975–1995, it is not obvious for 1995–2013, so no correction is made in the remainder of this study.

Having demonstrated that sunspot-based SBW estimates can be used to make a first-order reconstruction of the global solar wind structure of the period 1975–2013, we now extend the reconstruction back through the 17^th^ century. Because of “spin up” of the OSF reconstruction and associated SBW estimate, the reconstructions presented here starts in 1617 (rather than 1611), as results from this date onwards do not depend on chosen initial conditions.

**Figure 1:**
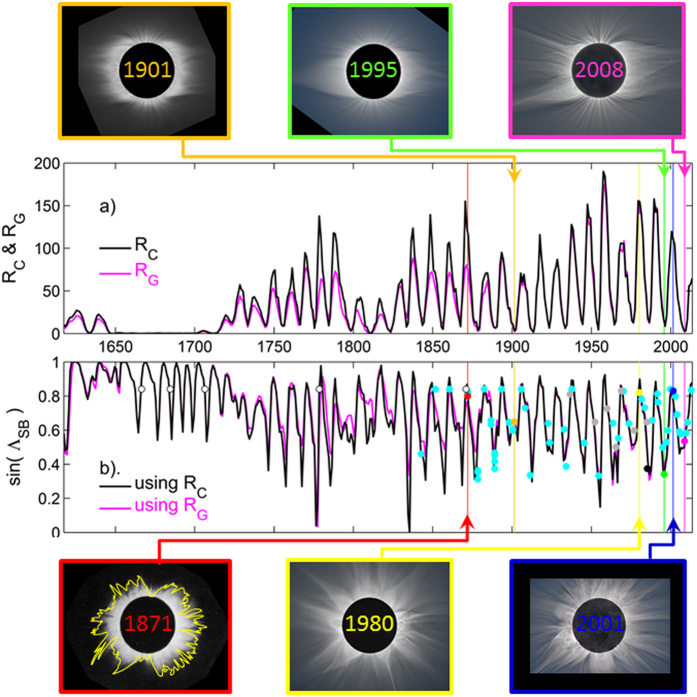
Annual variations in (**a**) corrected (R_C_, in black) and group (R_G_, in pink) sunspot numbers and (**b**) the sine of the modelled streamer-belt width (SBW) from R_C_ and R_G_^27^. Circles in (**b**) are scaled from the eclipse images listed, with sources, in Appendix A. Coloured points and lines relate to the selected example events for sunspot minimum/maximum shown along the top/bottom of the plots. Light blue dots are SBW from photographs and detailed paintings or lithographs made from photographic images; open circles are from descriptive texts and sketches, the black dot is from a Skylab coronagraph image and the grey points are from the Loucif and Koutchmy^52^ catalogue.

**Figure 2:**
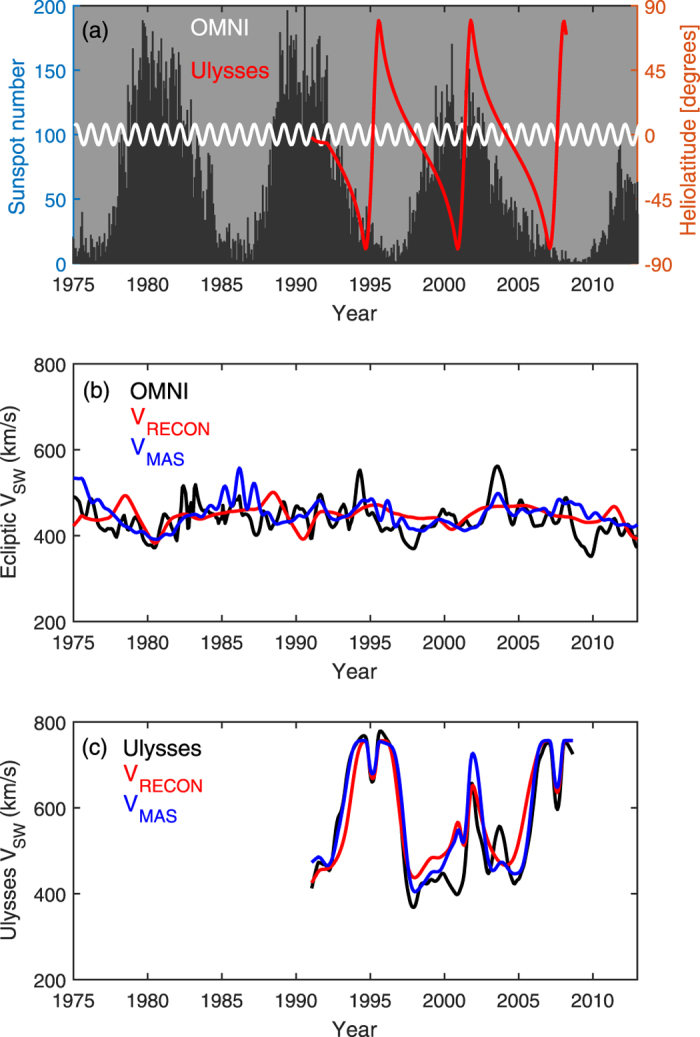
(**a**) The black-shaded region and left-hand axis show monthly sunspot number over the period 1975–2013. The right-hand axis, and red and white curves show the heliolatitude of the Ulysses and OMNI near-Earth spacecraft, respectively. (**b**) 1-year rolling means of solar wind speed in near-Earth space observed by the OMNI spacecraft (black), predicted by the MAS model (V_MAS_, blue) and the sunspot-based reconstruction (V_RECON_, red). (**c**) The same as panel (**b**), but for the Ulysses spacecraft.

**Figure 3:**
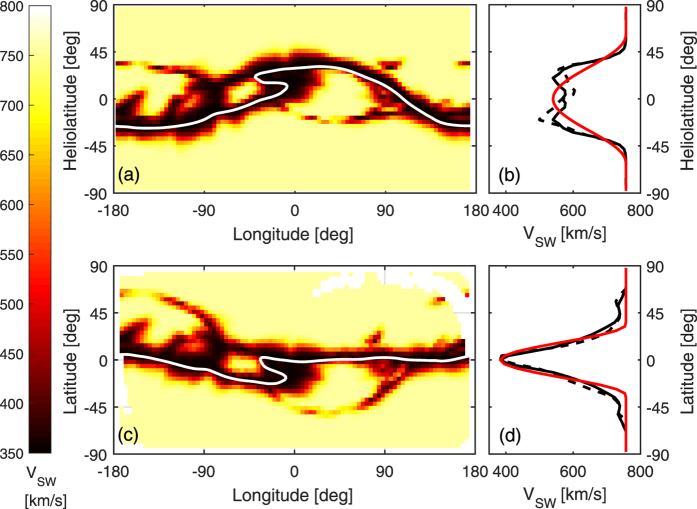
A summary of the solar wind speed from the MAS model, V_MAS_, for Carrington rotation 1756 (December 1984). Panels a and b show V_MAS_ in heliographic coordinates, whereas panels c and d show V_MAS_ inclined to maximise the difference in speed between the poles and equator. Panels a and c show latitude-longitude maps of V_MAS_ at 30 Rs. The HCS is shown as a thin white line. Panels b and d show zonal means of V_MAS_ as a black dashed line. The solid black line shows an average across north and south hemispheres. The red line shows the best functional fit, as described in the text.

**Figure 4:**
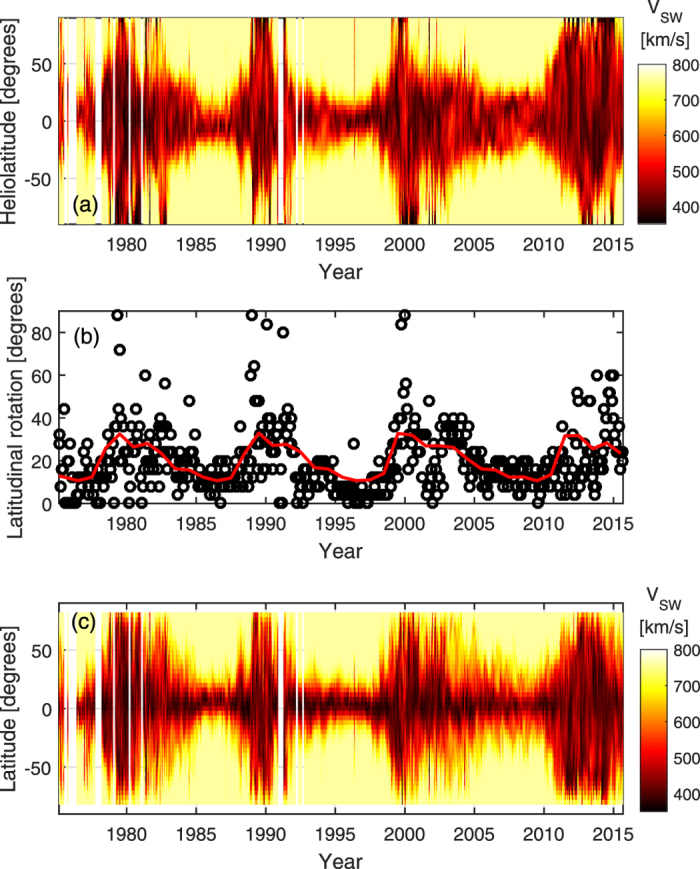
Summary of V_MAS_ over Carrington rotations 1625 to 2168 (i.e., Feb 1975 to Sept 2015). Panel (a) shows zonal means of V_MAS_ as a function of heliographic latitude and time. Panel (b) show the angular inclination required to maximise the difference in zonal mean V_MAS_ between the poles and equator for each Carrington rotation (black circles). The red line shows an annual resolution mean over all three solar cycles. Panel (c) shows the same as panel (a), but in the inclined coordinate system.

**Figure 5:**
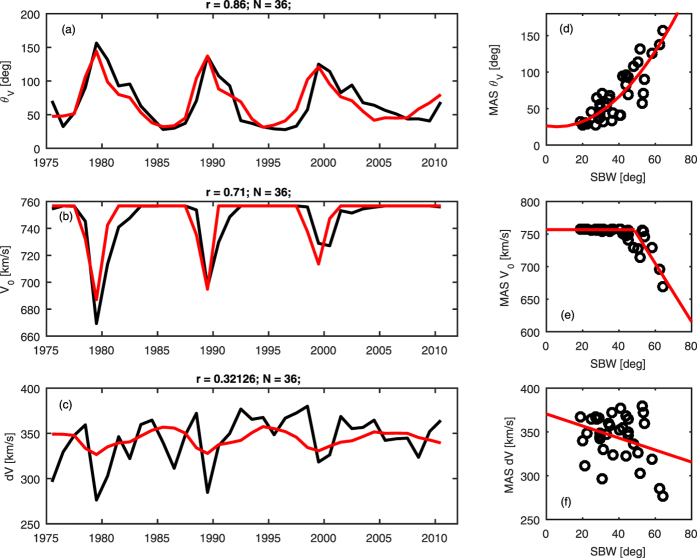
Panels (a) to (c) show time series of the angular width of slow wind band (θ_W_), the maximum solar wind speed (V_0_) and the depth of the slow wind band (dV), respectively, for MAS solutions (black) and reconstructed from sunspot-based estimates of streamer belt width (red). Panels (d) to (f) show scatter plots of these parameters from MAS with the sunspot-based estimates of streamer belt width (SBW). Red lines show the best fits, described in the text.

**Figure 6:**
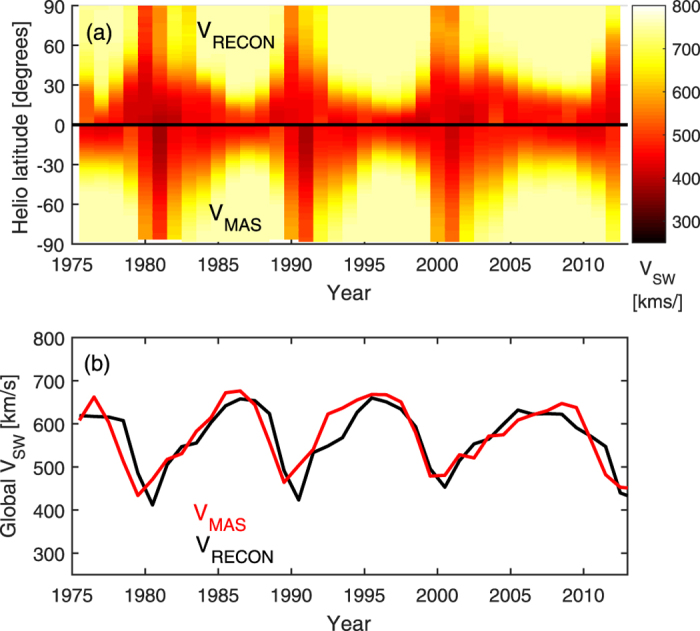
Comparison of annual means of solar wind speed from MAS (V_MAS_) and the reconstructed from the sunspot-based estimates of streamer-belt width (V_RECON_) over the period 1975–2013. (**a**) Zonal-mean solar wind speed as a function heliographic latitude and time for V_RECON_ (top) and V_MAS_ (bottom). (**b**) Global mean (i.e., latitudinally integrated, allowing for the reduced element area towards the poles) V_MAS_ (red) and V_RECON_ (black).

**Figure 7:**
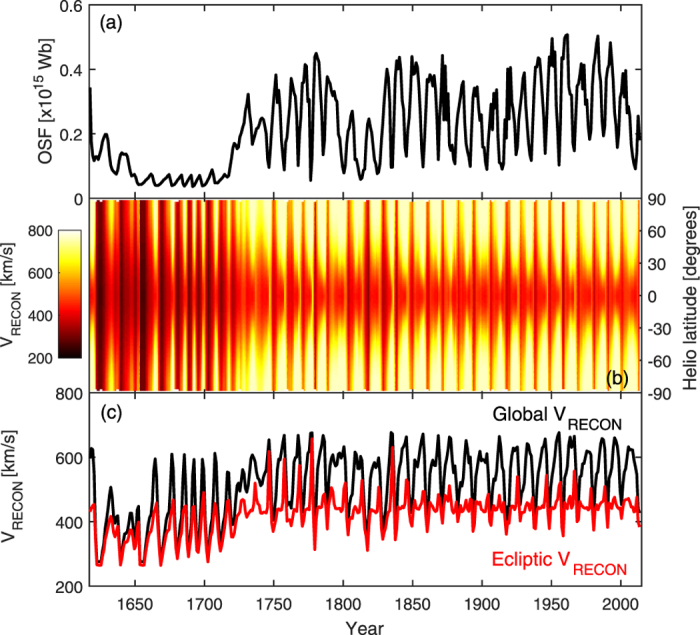
Annual means of sunspot-based reconstructions of the global solar wind (V_RECON_) over the period 1617 to 2013. (**a**) Unsigned open solar flux, OSF. (**b**) Zonal mean V_RECON_ as a function of heliographic latitude and time. (**c**) Global mean (black) and in-ecliptic (red) values of V_RECON_.

Figure 7a shows the (unsigned) open solar flux, OSF, estimated from sunspot observations^36^, from which the SBW (Fig. 1b) is subsequently computed^27^. The Maunder minimum (MM, approximately 1650 to 1710) and, to a lesser extent, the Dalton minimum (approximately 1795 to 1815), are identifiable as periods of reduced OSF. Panel b shows the zonal mean V_RECON_ as a function of heliographic latitude and time, while panel c shows global average (black) and ecliptic (red) values. For ~1750–2013, global V_RECON_ shows a strong solar cycle variation, with values around 600 km/s and short dips to approximately 450 km/s at solar maximum. There is little long-term variation over this period, though the Dalton minimum shows a slight decrease in the peak (i.e., solar minimum) V_RECON_. Over the period 1850–2013, the in-ecliptic V_RECON_ is fairly constant around 420 km/s, with only small (50–70 km/s), short-duration deviations. This is in rough agreement with near-Earth spacecraft measurements over the period 1963–2015 and values reconstructed from geomagnetic activity after 1845^38^. During 1750–1850, there are slightly larger but still short-lived in-ecliptic solar wind speed deviations. During and prior to the MM both global and in-ecliptic V_RECON_ drop substantially. The form of the solar cycle variation of global V_RECON_ is also different during the MM, with nominal values of around 350 km/s and short-duration upward spikes to approximately 600 km/s (i.e., there is a change from a solar minimum to solar maximum dominated heliosphere). The amplitude of the solar cycle variation of in-ecliptic V_RECON_ is also enhanced at this time, with a typical variation from 270 to 430 km/s. It’s likely the change in behaviour during the MM is a result of the switch from source-driven (i.e., sunspot) variations in OSF, to loss-driven variations, which has been shown to alter phase of the solar cycle variation in OSF during the MM^39^. Perhaps most interesting is the period 1617–1650, which contains small amplitude sunspot cycles just prior to the MM (see also Fig. 1a). Here, the global V_RECON_ drops to its lowest values, despite the OSF being enhanced above MM values. This is discussed further in the next section.

**Figure 8:**
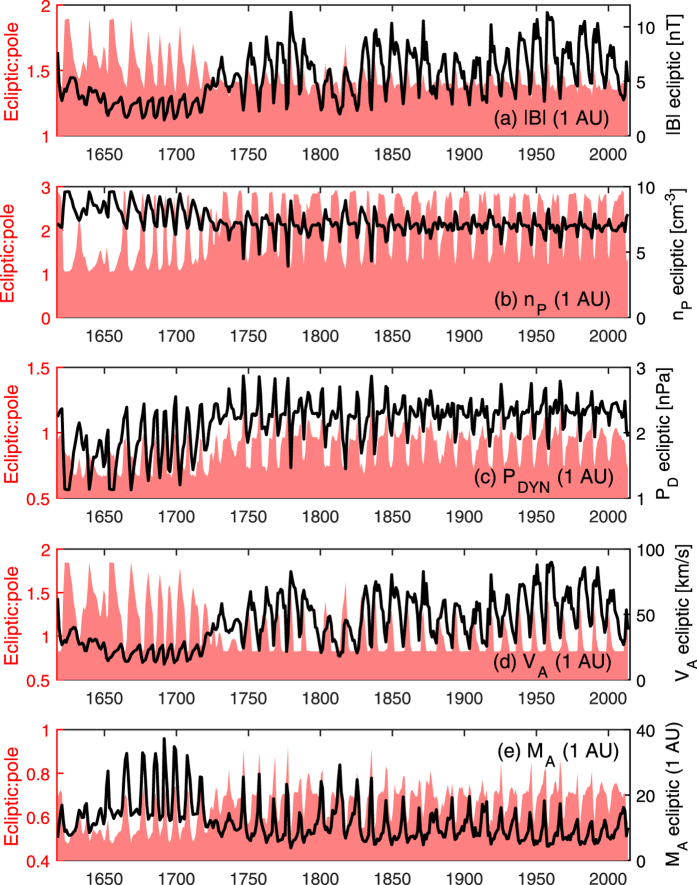
Reconstructed annual solar wind properties at 1 AU, for the period 1617–2013. Black lines and right-hand axes show in-ecliptic values, pink-shaded regions and left-hand axes show the ratio of ecliptic to polar values. Panels show (**a**) the magnetic field intensity, |B|; (**b**) the proton density, n_P_; (**c**) the dynamic or “ram” pressure, P_D_; (**d**) the Alfven speed, V_A_; and (**e**) the Alfven Mach number, M_A_, of the solar wind.

These new estimates of the global V_SW_ enable a number of further heliospheric parameters to be calculated on both a theoretical basis and by assuming empirical relations between parameters established from spacecraft data are valid over the prior four centuries. The black line in Fig. 8a shows the in-ecliptic magnetic field intensity, |B|, at 1 AU using the radial magnetic field magnitude determined from OSF (Fig. 7a) and assuming a Parker spiral angle consistent with the in-ecliptic solar wind speed. As expected, the variation closely follows the OSF, though the fractional variation between the MM and modern era is lower in |B| than in OSF, as the reduced V_SW_ during the MM increases the winding of the Parker spiral, adding to |B|. The pink-shaded area in Fig. 8a shows the ratio of ecliptic |B| to polar |B| at 1 AU, again assuming an ideal Parker spiral. For 1730–2013, this ratio is fairly constant at around 1.4:1, but during the MM the slower solar wind speeds cause an over-winding of the ecliptic HMF and hence an increase in the ecliptic |B|, resulting in a higher ratio of nearly 2:1.

Proton temperature, T_P_, can be computed from V_SW_ using empirical relations^40^, assuming they are unchanged during grand minima. Similarly, the assumption of constant mass flux in the solar wind^41^, can be used to estimate proton density, n_P_. Taking 27-day means of the entire OMNI, HELIOS and Ulysses datasets, we find a good linear anti-correlation (r = −0.69 for N = 567) between observed n_p_ (scaled to a radial distance of R_1_ = 1 AU. i.e., n_P_/R_1_^2^) and V_SW_. As the data are normally distributed, we use an ordinary least squares fit to to determine n_P_ (R_1_) = −0.0146 V_SW_ [km/s] + 13.7 cm^−3^. Using this relation, Fig. 8b shows the reconstructed annual n_P_ at 1 AU, in the same format as panel a. As expected from the linearity of the n_P_ - V_SW_ relation used, reconstructed n_P_ shows the same qualitative features as V_SW_: relatively little in-ecliptic variation for 1730–2013, but a marked change during the MM, in this case an increase of ~40%. The n_P_ reconstruction allow us to quantitatively investigate dynamic pressure (P_D_, which varies as n_P_ V_SW_^2^), Alfven speed (V_A_) and Alfven Mach number (M_A_) variations over 1617–2013. These are shown by panels c, d and e, respectively. During the MM, the in-ecliptic P_D_ is reduced by more than a factor 2 over the modern era. The in-ecliptic V_A_ variation closely follows the B variation and hence is suppressed during the MM. Finally, the in-ecliptic M_A_ increases by up to a factor 4 during the MM compared to the modern era.

## Discussion and Conclusions

In this study, we have used sunspot-based reconstructions of open solar flux (OSF) and streamer belt width (SBW) to provide the first estimate of global solar wind conditions over the last 400 years. Calibration was performed using 35 years of output from a photospheric magnetic field-constrained global magnetohydrodynamic (MHD) model of the corona and solar wind. The reconstructed annual solar wind speed (V_SW_) agrees well with both *in situ* spacecraft observations and the global solar wind speed inferred from the MHD model. Assuming these relations hold prior to the space age, annual solar wind speed reconstructions are extended back to 1617. A dipolar solar wind structure (fast/slow wind from the poles/equator) has dominated for much of the last 300 years, with short intervals of slow wind extending to all latitudes around solar maximum. Paradoxically, during the Maunder minimum (MM, 1650–1710), solar maximum-like conditions dominated, with fast wind over the poles for shorter durations and over a reduced latitudinal range than for the last 300 years. This, however, agrees well with the “most probable” MM coronal magnetic field configuration considered by Riley, *et al*.^16^. In-ecliptic, the annual zonal-mean solar wind speed varies very little (~70 km/s variations about a mean value of 420 km/s) back to circa 1715, but drops to a minimum of approximately 275 km/s during and prior to the MM, in approximate agreement with estimates by Cliver and von Steiger^42^. At the times of extremely low in-ecliptic V_SW_, polar V_SW_ is similarly reduced, suggesting any longitudinal structure in in-ecliptic V_SW_ is likely to be very weak. The longest periods of sustained slow wind actually occur prior to the Maunder minimum, when there were weak sunspot cycles. This appears to be the result of critical balance between OSF source and loss terms^39^ which results in maximum loss of coronal-hole flux compared to production of streamer-belt flux, maximising SBW.

From the OSF and V_SW_ reconstructions, it is possible to further estimate: magnetic field intensity (B) by assuming a Parker spiral heliospheric magnetic field; solar wind proton density (n_P_) by assuming constant mass flux; and solar wind proton temperature (T_P_) using empirical relations. These data are made available as Supplementary Material. From these parameters, we further derive values for the solar wind dynamic pressure (P_DYN_), Alfven speed (V_A_) and Alfven Mach number (M_A_). The full implications of these results will be investigated in detail in a number of follow-on studies. Here, we discuss the first-order implications. While the MM specifically is discussed below, it is expected that these findings could be broadly applied to any past or future^18^ grand minima of solar activity.

Firstly, we consider the terrestrial implications. Space weather is primarily the result of rapid changes in the space environment, rather than annual variations reconstructed in this study. Nevertheless, the equilibrium state of the terrestrial magnetospheric system is expected to be very different under MM than modern conditions. This, in turn, will mean a different response to a space weather driver, such as a fast coronal mass ejection. Future work will use a global MHD model of the coupled magnetosphere-ionosphere-thermosphere system to quantitatively investigate this. But even without a numerical model it is possible to draw some qualitative conclusions. The lower P_DYN_ during the MM would increase the average stand-off distance of the dayside magnetopause^43^. The width of the far magnetospheric tail, however, is controlled by the solar wind static pressure, P_STA_ = n_p_kT_SW_ + B^2^/(2μ_o_). As the higher n_p_ and T_P_ have a larger effect on P_STA_ than the reduction in B, the tail would, on average, be somewhat thinner during the MM than in modern times. Thus the magnetosphere would have presented a smaller cross-sectional area to the solar wind, reducing the electric field placed across it by the solar wind and the total solar wind energy that it intersects. A reduction in V_SW_ and B would mean a reduction in the solar wind electric field, which in turn would combine with the smaller diameter of the magnetosphere to reduce the trans-polar cap potential and polar cap area^44^. Thus the Earth’s magnetosphere would have been somewhat more Jupiter-like, with the part driven by solar wind-driven convection smaller in extent, and the part driven by internal dynamics and co-rotation larger in volume. In addition to an expected reduction in both recurrent and non-recurrent geomagnetic storms during the MM, the expected poleward motion of the nominal auroral oval position may further help explain the dearth of auroral reports from that period for all but the most northerly locations^15^. Beyond the magnetopause, the enhanced M_A_ suggests that the bow shock strength would be enhanced, resulting in more efficient energetic particle acceleration, while the bow shock stand-off distance would be increased on average, resulting in a thicker magnetosheath^45^.

Secondly, we consider the implications for the global heliosphere. Again, a future study will use the reconstructed solar wind parameters with a MHD model of the global heliosphere, but here we consider the first-order implications. Most obviously, a drop in P_DYN_ will result in an overall smaller heliosphere, though the contribution of pick-up ions to the total solar wind momentum budget^46^ means the P_DYN_ decrease at large heliocentric distances will be lower than the factor 2 between modern and MM 1-AU values. Any calculation of the heliopause distance under grand solar minima conditions will also need to account for the change in pick-up ion acceleration under the MM reduction in B, particularly out of the ecliptic plane. The shape of the heliosphere is also likely change under MM conditions. For the modern era, P_DYN_ has been ~2–3 higher at the poles than the solar equator^47^, which results in latitudinal asymmetry in the heliopause stand-off distance and termination shock location^48^. During much of the MM, however, P_DYN_ becomes almost uniform with latitude for a greater period of time, suggesting a more spherical heliosphere and termination shock.

In turn, there will also be a number of implications for cosmic ray intensity in near-Earth space, with potential knock-on effects for long-term heliospheric reconstructions on the basis of cosmogenic radionuclide records in ice cores and tree trunks^23^,^49^,^50^. The relative abundance of radioisotopes such as ^10^Be and ^14^C can be used to determine the effective shielding of heliosphere from the interstellar cosmic ray spectrum, referred to as the heliospheric modulation potential. Interpreting the modulation potential in terms of heliospheric parameters, such as OSF, necessitates a number of assumptions about the size of the heliosphere, the solar wind speed and the scaling of cosmic ray scattering centers with the HMF intensity^20^. During grand minima, all of these properties will change, to some degree. As already discussed, we expect a smaller heliosphere, with lower and more symmetric solar wind speeds. The lack of latitudinal solar wind speed structure suggests reduced corotating interaction region formation and hence reduced cosmic ray scattering (even for the same OSF). Furthermore, we note that enhanced V_A_ during the MM would increase the termination shock strength and may affect the efficiency of anomalous cosmic ray acceleration^46^. While the effect of changing size/shape of the heliosphere is expected to be small on GeV (and greater) energy particles which are largely responsible for cosmogenic isotope production, and hence radionuclide reconstructions of the heliosphere and total solar irradiance^51^, it needs to be fully quantified via a galactic cosmic ray transport model and a cosmogenic isotope production model.

## Additional Information

**How to cite this article**: Owens, M. J. *et al*. Global solar wind variations over the last four centuries. *Sci. Rep.* **7**, 41548; doi: 10.1038/srep41548 (2017).

**Publisher's note:** Springer Nature remains neutral with regard to jurisdictional claims in published maps and institutional affiliations.

## Supplementary Material

Supplementary Materials

Supplementary Dataset 1A

Supplementary Dataset 1B

Supplementary Dataset 1C

Supplementary Dataset 1D
