# Supplementary material for: Global solar wind variations over the last four centuries
Source: Sci Rep. 2017 Jan 31;7:41548. doi: 10.1038/srep41548 (PMC5282500; doi:10.1038/srep41548)
Supplement: Supplementary Materials [file srep41548-s1.doc]

**Global solar wind variations over the last four centuries**

M.J. Owens^1^, M. Lockwood^1^, P. Riley^2^

^1^Space and Atmospheric Electricity Group, Department of Meteorology, University of Reading, Earley Gate, PO Box 243, Reading RG6 6BB, UK

^2^Predictive Science Inc., 9990 Mesa Rim Rd, Suite 170, San Diego, CA 92121, USA

Supplementary materials: Eclipse images used in Figure 1.

Owens, Lockwood and Riley (2016) Eclipse Images Used

Type codes are: 1. Reprocessed photograph; 2. Un-reprocessed photograph; 3. Drawing/painting/lithograph from photographs; 4. Coronograph Image; 5. Value Scaled by Loucif and Koputchmy (1989); 6. Written report

| year | mn | day | [Λ_SB_]_E_  (deg.) | [Λ_SB_]_W_  (deg.) | code | Imaged from | Source | Reference | # |
| --- | --- | --- | --- | --- | --- | --- | --- | --- | --- |
| 2013 | 11 | 03 | 90.0 | 90.0 | 1 | Pokwero, Uganda | Miloslav Druckmüller, Brno Univ. | http://www.zam.fme.vutbr.cz/~druck/eclipse/ | 1 |
| 2012 | 11 | 13 | 86.1 | 85.5 | 1 | Cairns,_Australia | Miloslav Druckmüller, Brno Univ. | http://www.zam.fme.vutbr.cz/~druck/eclipse/ | 2 |
| 2010 | 07 | 11 | 58.4 | 71.9 | 1 | Tatakoto Atoll, French Polynesia | Miloslav Druckmüller, Brno Univ. | http://www.zam.fme.vutbr.cz/~druck/eclipse/ | 3 |
| 2009 | 07 | 22 | 64.8 | 57.9 | 1 | Enewetak Atoll, Marshall Islands | Miloslav Druckmüller, Brno Univ. | http://www.zam.fme.vutbr.cz/~druck/eclipse/ | 4 |
| 2008 | 08 | 01 | 58.2 | 59.3 | 1 | South-Western Mongolia | Miloslav Druckmüller, Brno Univ. | http://www.zam.fme.vutbr.cz/~druck/eclipse/ | 5 |
| 2006 | 03 | 29 | 59.9 | 62.2 | 1 | Göreme, Cappadocia, Turkey | Miloslav Druckmüller, Brno Univ. | http://www.zam.fme.vutbr.cz/~druck/eclipse/ | 6 |
| 2005 | 04 | 08 | 65.1 | 58.6 | 1 | aboard ‘Galapagos Legend’ in Pacific | Miloslav Druckmüller, Brno Univ. | http://www.zam.fme.vutbr.cz/~druck/eclipse/ | 7 |
| 2003 | 11 | 23 | 68.9 | 67.8 | 1 | Aircraft over Antarctica | Miloslav Druckmüller, Brno Univ. | http://www.zam.fme.vutbr.cz/~druck/eclipse/ | 8 |
| 2002 | 12 | 04 | 74.1 | 82.8 | 1 | Messina, South Africa | Miloslav Druckmüller, Brno Univ. | http://www.zam.fme.vutbr.cz/~druck/eclipse/ | 9 |
| 2001 | 06 | 21 | 84.4 | 84.4 | 1 | Sumbe, Angola | Miloslav Druckmüller, Brno Univ. | http://www.zam.fme.vutbr.cz/~druck/eclipse/ | 10 |
| 1999 | 08 | 11 | 90.0 | 90.0 | 1 | Németkér_Hungary | Miloslav Druckmüller, Brno Univ. | http://www.zam.fme.vutbr.cz/~druck/eclipse/ | 11 |
| 1998 | 02 | 06 | 59.7 | 64.7 | 1 | Maracaibo, Venezuela | Miloslav Druckmüller, Brno Univ. | http://www.zam.fme.vutbr.cz/~druck/eclipse/ | 12 |
| 1997 | 03 | 09 | 48.4 | 68.5 | 1 | Yerofei Pavlovich, Siberia, Russia | Miloslav Druckmüller, Brno Univ. | http://www.zam.fme.vutbr.cz/~druck/eclipse/ | 13 |
| 1995 | 10 | 24 | 56.0 | 43.3 | 1 | Nim Ka Thana, Rajastan, India | Miloslav Druckmüller, Brno Univ. | http://www.zam.fme.vutbr.cz/~druck/eclipse/ | 14 |
| 1994 | 11 | 03 | 65.7 | 48.0 | 1 | Sevaruyo, Bolivia | Miloslav Druckmüller, Brno Univ. | http://www.zam.fme.vutbr.cz/~druck/eclipse/ | 15 |
| 1991 | 07 | 11 | 39.2 | 122.4 | 1 | Baja, California, USA | Miloslav Druckmüller, Brno Univ. | http://www.zam.fme.vutbr.cz/~druck/eclipse/ | 16 |
| 1990 | 22 | 07 | 95.6 | 53.6 | 1 | Markovo, Chukotka, Russia | Miloslav Druckmüller, Brno Univ. | http://www.zam.fme.vutbr.cz/~druck/eclipse/ | 17 |
| 1988 | 03 | 18 | 59.7 | 72.1 | 1 | General Santos City, Philippines | High Altitude Observatory, USA | http://mlso.hao.ucar.edu/hao-eclipses.php | 18 |
| 1985 | 03 | 11 | 47.3 | 54.8 | 4 | Solar Maximum Mission Satellite | Bird, M. K. & P. Edenhofer (1990) *Remote Sensing Observations of the Solar Corona* | in *Physics of the Inner Heliosphere I, XI*, p. 13., Springer-Verlag, Berlin, Germany. eds. R. Schewenn and E. Marsch | 19 |
| 1984 | 11 | 22 | 65.2 | 65.2 | 5 |  | Loucif M.L. & S. Koputchmy (1989)  *Solar cycle variations of coronal structures* | Astron. Astrophys. Suppl. Series, 77, 45-66.  Solar cycle variations of coronal structures, Astron. Astrophys. Suppl. Series, 77, 45-66. 20 | 20 |
| 1983 | 06 | 11 | 79.6 | 63.5 | 1 | Tanjung Kodok, Java, Indonesia | High Altitude Observatory, USA | http://mlso.hao.ucar.edu/hao-eclipses.php | 21 |
| 1981 | 07 | 31 | 59.7 | 93.1 | 1 | Tarma, Siberia, Russia | High Altitude Observatory, USA | http://mlso.hao.ucar.edu/hao-eclipses.php | 22 |
| 1980 | 02 | 16 | 82.2 | 82.2 | 1 | India | High Altitude Observatory, USA | http://mlso.hao.ucar.edu/hao-eclipses.php | 23 |
| 1976 | 10 | 23 | 62.2 | 62.3 | 5 |  | Loucif M.L. & S. Koputchmy (1989)  *Solar cycle variations of coronal structures* | Astron. Astrophys. Suppl. Series, 77, 45-66. | 24 |
| 1973 | 06 | 30 | 56.3 | 73.1 | 1 | Loiengalani, Kenya | High Altitude Observatory, USA | http://mlso.hao.ucar.edu/hao-eclipses.php | 25 |
| 1970 | 03 | 07 | 84.0 | 84.0 | 2 | San Carlos Yautepec, Mexico | High Altitude Observatory, USA | http://mlso.hao.ucar.edu/hao-eclipses.php | 26 |
| 1968 | 10 | 22 | 84.5 | 84.5 | 5 |  | Loucif M.L. & S. Koputchmy (1989)  *Solar cycle variations of coronal structures* | Astron. Astrophys. Suppl. Series, 77, 45-66. | 27 |
| 1966 | 11 | 12 | 56.5 | 62.7 | 2 | Pulacayo, Bolivia | High Altitude Observatory, USA | http://mlso.hao.ucar.edu/hao-eclipses.php | 28 |
| 1965 | 05 | 30 | 57.0 | 57.0 | 5 |  | Loucif M.L. & S. Koputchmy (1989)  *Solar cycle variations of coronal structures* | Astron. Astrophys. Suppl. Series, 77, 45-66. | 29 |
| 1963 | 07 | 20 | 63.6 | 66.1 | 2 | Sourdough, Alaska | High Altitude Observatory, USA | http://mlso.hao.ucar.edu/hao-eclipses.php | 30 |
| 1959 | 10 | 02 | 71.0 | 71.0 | 5 |  | Loucif M.L. & S. Koputchmy (1989)  *Solar cycle variations of coronal structures* | Astron. Astrophys. Suppl. Series, 77, 45-66. | 31 |
| 1954 | 06 | 30 | 47.2 | 51.4 | 5 |  | Tlatov, A.G. (2010) *The centenary variations in the solar corona shape in accordance with the observations during the minimal activity epoch* | Astron. & Astrophys. 522, A27.  doi: 10.1051/0004-6361/201014082 | 32 |
| 1952 | 02 | 25 | 65.2 | 65.3 | 2 |  | Loucif M.L. & S. Koputchmy (1989)  *Solar cycle variations of coronal structures* | Astron. Astrophys. Suppl. Series, 77, 45-66. | 33 |
| 1945 | 06 | 09 | 65.2 | 51.2 | 3 |  | Tlatov, A.G. (2010) *The centenary variations in the solar corona shape in accordance with the observations during the minimal activity epoch* | Astron. & Astrophys. 522, A27.  doi: 10.1051/0004-6361/201014082 | 34 |
| 1937 | 08 | 06 | 90.0 | 90.0 | 2 | Canton island | High Altitude Observatory, USA | http://mlso.hao.ucar.edu/hao-eclipses.php | 35 |
| 1936 | 06 | 19 | 80.5 | 80.5 | 5 |  | Loucif M.L. & S. Koputchmy (1989)  *Solar cycle variations of coronal structures* | Astron. Astrophys. Suppl. Series, 77, 45-66. | 36 |
| 1934 | 01 | 14 | 50.8 | 52.6 | 3 |  | Tlatov, A.G. (2010) *The centenary variations in the solar corona shape in accordance with the observations during the minimal activity epoch* | Astron. & Astrophys. 522, A27.  doi: 10.1051/0004-6361/201014082 | 37 |
| 1932 | 08 | 31 | 63.1 | 62.2 | 2 | location unknown | High Altitude Observatory, USA | http://mlso.hao.ucar.edu/hao-eclipses.php | 38 |
| 1923 | 09 | 10 | 48.2 | 68.5 | 3 |  | Tlatov, A.G. (2010) *The centenary variations in the solar corona shape in accordance with the observations during the minimal activity epoch* | Astron. & Astrophys. 522, A27.  doi: 10.1051/0004-6361/201014082 | 39 |
| 1922 | 09 | 21 | 64.9 | 64.6 | 2 | Wallal, Australia | High Altitude Observatory, USA | http://mlso.hao.ucar.edu/hao-eclipses.php | 40 |
| 1911 | 04 | 28 | 51.3 | 47.5 | 3 |  | Tlatov, A.G. (2010) *The centenary variations in the solar corona shape in accordance with the observations during the minimal activity epoch* | Astron. & Astrophys. 522, A27.  doi: 10.1051/0004-6361/201014082 | 41 |
| 1908 | 03 | 01 | 74.5 | 68.2 | 2 | Flint Island | McLean, F.K. et al. (1909) *Report of the solar eclipse expedition to Flint Island January 3, 1908 : being an account of the observations made by the expedition under the leadership of F. K. McClean* | Archives of the Royal Society of London | 42 |
| 1905 | 08 | 30 | 90.0 | 90.0 | 2 | Alhama Spain | High Altitude Observatory, USA | http://mlso.hao.ucar.edu/hao-eclipses.php | 43A |
| 1905 | 08 | 30 | 90.0 | 90.0 | 3 | Guelma, Algeria, Daroca Spain and Porta Coeli, Spain (US Naval expedition) | Sketch by C.G. Abbot from various photographs | Abbot, C.G. (1911) *The Sun*, Appleton (New York and London) http://www.archive.org/stream/cu31924004973628#page/n239/mode/2up | 43B |
| 1901 | 05 | 18 | 63.6 | 67.1 | 1 | Padang, Sumatra | Judge, P.G., J. Burkepile, G. de Toma  M. Druckmuller (2010) *Historical eclipses and the recent solar minimum corona* | in *SOHO-23: Understanding a Peculiar Solar Minimum*, Astronomical Society of the Pacific Conference Series, 428, eds. S.R. Cranmer, J.T. Hoeksema, and J.L. Kohl | 44 |
| 1900 | 05 | 28 | 60.6 | 64.9 | 3 | Algieres | Maunder E.W. (1901) *The Total solar eclipse, 1900 : report of the expeditions organized by the British Astronomical Association to observe the total solar eclipse of 1900, May 28* | Archives of the Royal Society of London | 45A |
| 1900 | 05 | 28 | 62.0 | 62.4 | 2 | Thomaston, Georgia, USA | High Altitude Observatory, USA | http://mlso.hao.ucar.edu/hao-eclipses.php | 45B |
| 1898 | 01 | 22 | 67.0 | 63.0 | 2 | Jeur India | High Altitude Observatory, USA | http://mlso.hao.ucar.edu/hao-eclipses.php | 46A |
| 1898 | 01 | 22 | 67.5 | 62.8 | 3 | Jeur India | Lithograph by W.H. Wesley from Copeland’s photograph | Archive of the Royal Observatory, Edinburgh  Pang, A S.-K. (1994) *Victorian observing practices . printing and technology and representations of the solar corona (1): the 1860s and 1870s*, J. for the History of Astronomy, 249-274 | 46B |
| 1893 | 04 | 16 | 90.0 | 90.0 | 2 | Mina Bronces, Santiago, Chile | Contact print from the original glass plate negative. | Lick Observatory Plate Archive, Mt. Hamilton http://www.exploratorium.edu/eclipse/eclipse_photos5.html | 47A |
| 1893 | 04 | 16 | 90.0 | 90.0 | 2 | Mina Bronces, Santiago, Chile | High Altitude Observatory, USA | http://mlso.hao.ucar.edu/hao-eclipses.php | 47B |
| 1889 | 01 | 01 | 53.9 | 52.0 | 3 | Bartlett Springs, California, USA | sketch by W.H.M. Christie from the photograph plates by E.E. Barnard, in  Christie, W.H.M. (1889) *On the photographs of the corona at the solar eclipse of 1889, January* 1 | Monthly Notices of RAS (London), 49, (6), 343-352 | 48A |
| 1889 | 01 | 01 | 56.1 | 54.5 | 2 | Cayenne Fr. Guyana | High Altitude Observatory, USA | http://mlso.hao.ucar.edu/hao-eclipses.php | 48B |
| 1889 | 01 | 01 | 50.2 | 51.9 | 3 | Bartlett Springs, California, USA | Sketch by A. Hansky from plates by E.E. Barnard | Hansky, A. (1907) *Mittheilung der Nikolai-Haupt Sternearte zu Pulkovo*, 1, 81. Reproduced by Sampson, D.A. (1914)  *The Sun*, Cambridge University Press.  http://archive.org/stream/sunsamps00sampuoft#page/n5/mode/2up | 48C |
| 1889 | 01 | 01 | 53.5 | 55.4 | 2 | Norman, California, USA | Composite of negatives by F. Eigler | Various authors (1891) *A report of the observations made by the Washington University Eclipse Party at Norman California*, J Wilson and Sons, Cambridge, USA.  http://archive.org/stream/totaleclipseofsu00washrich#page/n7/mode/2up | 48D |
| 1886 | 08 | 29 | 64.4 | 64.2 | 3 | St. George, Grenada | L. Darwin, A. Schuster and E.W. Maunder (1889) *On the Total Solar Eclipse of August 29, 1886* | Phil. Trans. Royal Society of London. A, 180, 291-350 | 49A |
| 1886 | 08 | 29 | 61.3 | 70.4 | 3 | St. George, Grenada | Sketch by A. Hansky from plates by A. Schuster | Hansky, A. (1907) *Mittheilung der Nikolai-Haupt Sternearte zu Pulkovo*, 1, 81. Reproduced by Sampson, D.A. (1914)  *The Sun*, Cambridge University Press.  http://archive.org/stream/sunsamps00sampuoft#page/n5/mode/2up | 49B |
| 1882 | 05 | 17 | 90.0 | 90.0 | 3 | Sohag, Upper Egypt | Drawing by Mr. W.H. Wesley from the photographs by A. Schuster | Todd, M.L. (1894) *Total Eclipses of the Sun*, J. Wilson, Cambridge USA, 1894  http://archive.org/stream/totaleclipsesofs00todd#page/n9/mode/2up | 50A |
| 1882 | 05 | 17 | 90.0 | 90.0 | 3 | Sohag, Upper Egypt |  |  | 50B |
| 1878 | 05 | 29 | 44.088 | 52.899 | 3 | Central City, Colorado, USA | Sketch by A. Hansky from plates by O.L. Peers | Hansky, A. (1907) *Mittheilung der Nikolai-Haupt Sternearte zu Pulkovo*, 1, 81. Reproduced by Sampson, D.A. (1914)  *The Sun*, Cambridge University Press.  http://archive.org/stream/sunsamps00sampuoft#page/n5/mode/2up | 51A |
| 1878 | 05 | 29 | 48.9 | 52.2 | 3 | Creston, Wyoming, USA | Pastal drawing by Étienne Léopold Trouvelot from photographs | The Trouvelot astronomical drawings: Atlas  http://digitalgallery.nypl.org/nypldigital/id?trouvelot_003 | 51B |
| 1878 | 05 | 29 | 42.528 | 57.840 | 3 | Creston, Wyoming, USA | Sketch by W. Harkness | Todd, M.L. (1894) *Total Eclipses of the Sun*, J. Wilson, Cambridge USA, 1894  http://archive.org/stream/totaleclipsesofs00todd#page/n9/mode/2up |  |
| 1871 | 12 | 12 | 85.8 | 71.6 | 2 | Baikul, India | wet collodion photographic plate by Lord Lindsay | Archives of the Royal Greenwich Observatory  and http://www.astrosurf.com/re/history_astrophotography_timeline.pdf | 52A |
| 1871 | 12 | 12 | 81.5 | 78.7 | 3 | Baikul, India | Lithograph from photographs by W.H. Wesley | Pang, A S.-K. (1994) *Victorian observing practices . printing and technology and representations of the solar corona (1): the 1860s and 1870s*, J. for the History of Astronomy, 249-274 | 52B |
| 1871 | 12 | 12 | 83.5 | 82.1 | 3 | Baikul, India | Sketch by A. Hansky from plates by H. Davis | Hansky, A. (1907) *Mittheilung der Nikolai-Haupt Sternearte zu Pulkovo*, 1, 81. Reproduced by Sampson, D.A. (1914)  *The Sun*, Cambridge University Press.  http://archive.org/stream/sunsamps00sampuoft#page/n5/mode/2up | 52C |
| 1870 | 12 | 22 | 90.0 | 90.0 | 6 | Oran, Algeria | Sketch by Janssen | Todd, M.L. (1894) *Total Eclipses of the Sun*, J. Wilson, Cambridge USA, 1894  http://archive.org/stream/totaleclipsesofs00todd#page/n9/mode/2up | 53 |
| 1860 | 07 | 18 | 90.0 | 90.0 | 3 | Cujuli, Spain: see Hingley, P. D (2001) *The first photographic eclipse*, Astron. & Geophys., 42 (1), 1.18-1.23. doi: 10.1046/j.1468-4004.2001.0420011.18.x | Painting by Charles Pritchard and drawings by Sir Francis Galton from photographic plates by Warren de la Rue. | Plate 1 of *Sun, moon and stars: astronomy for beginners*, by A. Giberne (Seeley, London, 1910). Archives of the Royal Society of London  F. Galton (1861) *Vacation Tourists and Notes of Travel in 1860*, p.p434-444. McMillan, London  https://archive.org/details/cu31924092511223 | 54 |
| 1851 | 07 | 28 | 90.0 | 90.0 | 3 | Göttenburg, Christiania, and Christianstad, Sweden | Pencil drawing by G.B. Airy | Royal Greenwich Observatory archive, Airy Papers, Box 233, Fol. 214.  Also in: Rothermel, H. (1993) *Images of the Sun: Warren del la Rue, George Biddell Airy and celestial photography*, British Journal for the history of science, 26(2) 137-169 | 55 |
| 1842 | 07 | 08 | 55.0 | 55.0 | 6 | Perpignan, France | Sketch from plates by Arago F. (1851) Oeuvres complètes de François Arago, ed. J.-A. Barral, 13 vol. Gide et Leipzig, Weigel, Paris, t 7, pp 112-135. Clearly inaccurate near 1R_🞊_ so streamer belt width scaled directly from 2.5R_🞊_ instead of using usual regression | Todd, M.L. (1894) *Total Eclipses of the Sun*, J. Wilson, Cambridge USA, 1894  http://archive.org/stream/totaleclipsesofs00todd#page/n9/mode/2up | 56 |
| 1778 | 06 | 24 | 90.0 | 90.0 | 6 | Various | Various including one at sea by Don Antonio Ulloa. | Vaquero, J.M. (2003) *The Solar Corona in the Eclipse of 24 June 1778*, Solar Physics, 216 (1-2), 41-45  doi:10.1023/A:1026190412303 | 57 |
| 1706 | 05 | 12 | 90.0 | 90.0 | 6 | Marseilles, France | Report of a full solar “crown” by Laval and Chazelles | *An Abstract of a Letter Written from Geneva, May the 31th, 1706. N. S. by Monsieur J. Chr. Facio Duillier, R. S. S. to His Brother Mr Nic. Facio, R. S. S. Containing Some Observations of the Sun's Eclipse, on the 12th of May, 1706 NS.*  Phil Trans. Royal Society London, 25, 2241-2246  https://archive.org/details/jstor-102678 | 58A |
| 1706 | 05 | 12 | 90.0 | 90.0 | 6 | Montpellier, France | M.M. Plantade and Capié |  | 58B |
| 1683 | 01 | 27 | 90.0 | 90.0 | 6 | København, Denmark | Report by Olé (Olaus) Christensen Rømer of streamers all around the Sun “the circumference of solar disk was uneven because of the vapors” | All Rømer’s papers were destroyed by the great Fire of Copenhagen in 1728. Report from Le Monnier, P.C. (1741) *Histoire Céleste* | 59 |
| 1666 | 06 | 22 | 90.0 | 90.0 | 6 | Various | Reports of full “lunar” atmosphere in | *Observations Made in Several Places, of the Late Eclipse of the Sun, Which Hapned on the 22 of June, 1666,* Phil Trans. Royal Society London, 17, 295. 297 http://www.jstor.org/stable/101510 | 60 |
